# Endoreversible Modeling of a Hydraulic Recuperation System

**DOI:** 10.3390/e22040383

**Published:** 2020-03-26

**Authors:** Robin Masser, Karl Heinz Hoffmann

**Affiliations:** Institut für Physik, Technische Universität Chemnitz, 09107 Chemnitz, Germany; robin.masser@physik.tu-chemnitz.de

**Keywords:** non-equilibrium thermodynamics, endoreversible thermodynamics, energy recovery, compressible fluid, pressure losses, van der waals fluid, hydraulic systems

## Abstract

Hybrid drive systems able to recover and reuse braking energy of the vehicle can reduce fuel consumption, air pollution and operating costs. Among them, hydraulic recuperation systems are particularly suitable for commercial vehicles, especially if they are already equipped with a hydraulic system. Thus far, the investigation of such systems has been limited to individual components or optimizing their control. In this paper, we focus on thermodynamic effects and their impact on the overall systems energy saving potential using endoreversible thermodynamics as the ideal framework for modeling. The dynamical behavior of the hydraulic recuperation system as well as energy savings are estimated using real data of a vehicle suitable for application. Here, energy savings accelerating the vehicle around 10% and a reduction in energy transferred to the conventional disc brakes around 58% are predicted. We further vary certain design and loss parameters—such as accumulator volume, displacement of the hydraulic unit, heat transfer coefficients or pipe diameter—and discuss their influence on the energy saving potential of the system. It turns out that heat transfer coefficients and pipe diameter are of less importance than accumulator volume and displacement of the hydraulic unit.

## 1. Introduction

Commercial vehicles are subject to frequent velocity changes during their operation especially considering regional and distribution transport in urban area. The recovery of kinetic energy during braking or downhill driving combined with propulsion support can therefore tremendously reduce fuel consumption and thus operating costs and pollutant emissions. Recuperation systems designed to accomplish that are based on energy storage devices which absorb the kinetic energy of the vehicle to a high degree and as efficiently as possible and are able to reuse stored energy at a later time. This energy can be used for either propulsion support and the operation of auxiliary equipment such as tilting or depositing devices and loading cranes. For charging and discharging the storage devices—whether based on electrical, mechanical or hydraulic technology—energy converters adapted to the respective technology are required which also provide a possibility for an emission-free operation of the auxiliary equipment.

Electric and mechanical recuperation systems—or hybrid drive systems—have been extensively studied in the last decades [1,2,3,4,5]. Hydraulic hybrid systems have also been part of a number of studies [6,7,8,9,10], as well as their individual components such as the hydraulic accumulator [11,12,13]. However, the focus of those investigations was rather on the appropriate control strategies for different hybrid types than on an overall thermodynamic description of the system. To investigate which effects may occur and how they can influence the recuperation potential of the system, such a comprehensive thermodynamic description is derived in this work. Based on real driving data, we then use this detailed model to predict the energy saving potential as well as the influence of certain design parameters and thermal effects.

The modeling framework for the desired comprehensive thermodynamic description can in general be chosen at different levels of detail and complexity. Thermodynamic descriptions can range from the simple level of classical equilibrium thermodynamics through classical irreversible thermodynamics and extended irreversible thermodynamics (EIT) to kinetic theory and beyond. With increasing complexity, such continuum approaches use enlarged state spaces; for instance, EIT uses the heat and entropy fluxes as independent variables leading to generalizations of entropy for this out of equilibrium description [14,15,16]. These continuum approaches and others [17,18,19] lead to extremely expensive numerical solution requirements for the given spatial design of realistic recuperation systems and are thus not practical.

The modeling framework we use here instead is endoreversible thermodynamics—a finite-time thermodynamics approach based on subdividing the overall system into discrete reversible acting subsystems which are linked by reversible and irreversible energy transports [20]. This ansatz is able to combine equilibrium thermodynamics knowledge with irreversible phenomenological or theoretical relationships, thus leading to a quantification of the *always* positive entropy production in the transport between the subsystems. It has proven to be a suitable tool for modeling complex and dissipative systems while providing reliable predictions. Using endoreversible thermodynamics technical and natural systems of different types have been modeled such as heat engines [21,22,23,24,25,26,27,28,29,30], distillation processes [31], thermoelectric generators [32,33,34], electrochemical devices [35,36] and solar thermal heat engines [37,38]. In addition, the effects of stochastic fluctuations were recently modeled using this ansatz [39,40,41].

A hydraulic recuperation system was modeled using endoreversible thermodynamics to investigate the influence of dissipative heat transfers within the hydraulic accumulator by Schwalbe et al. [42]. For this first model, however, simplifying assumptions have been made that may neglect some relevant effects regarding the performance of the overall system. In addition to the extension of endoreversible formalism to chemical reactors, Wagner et al. introduced multi-extensity fluxes [43]. These interactions transferring two or more coupled extensive quantities are essential for the description of many particle or fluid transports occurring in reality. Hence, we incorporate these multi-extensity fluxes to obtain a more detailed modeling of the hydraulic fluid flows within the recuperation system. Another recent expansion to endoreversible thermodynamics is a dissipative engine setup based on a more general interaction description by Masser et al. [44]. This dissipative engine setup is suitable to easily and consistently incorporate engines with given performance characteristics—such as measured efficiency or power output data—into an endoreversible description without having to build a complex model with the irreversible processes occurring within the engine in order to reproduce named performance characteristics. This is especially useful for large composite systems such as the hydraulic recuperation system considered here. We use this engine setup to model the hydraulic engine in order to focus rather on mutual influences between the individual hydraulic components than on the modeling of this particular engine itself.

This work serves as an example of how endoreversible thermodynamics can be applied to composite and hydraulic systems and as a preliminary investigation for a possible practical application. The purpose of this manuscript is thus to discover the general trends and effects occurring within the system in question rather than to obtain an accurate simulation of it.

We start with a brief outline of endoreversible formalism in the following section. In Section 3, the functioning of the hydraulic recuperation system is explained and the modeling of the individual components as well as the composite system is described. After that, simulation results, especially with regard to energy savings and the influence of selected parameters, are presented.

## 2. Endoreversible Formalism

The approach of endoreversible thermodynamics [20,45] is to divide the system in question into reversible acting subsystems and to describe the processes taking place by means of reversible or irreversible interactions. In subsystems, a distinction is made between finite and infinite reservoirs as well as engines that only serve for energy conversion. Typically, a finite reservoir *i* is described by its energy $E_{i}$, which is a function of its extensities. For each extensity $X_{i}^{\alpha }$, there is a corresponding intensity $Y_{i}^{\alpha }$ that be obtained by

(1) $$Y_{i}^{\alpha }=\frac{\partial E_{i}\left(X_{i}^{\alpha }\right)}{\partial X_{i}^{\alpha }}.$$

The superscript α is used to define the specific extensity. For instance, if α=S, then the extensity $X^{S}$ is the entropy *S* and the corresponding intensity $Y^{S}$ is the temperature *T*. Accordingly, an energy change of subsystem *i* can be written as the sum of changes in extensities times the corresponding intensities of the subsystem

(2) $$dE_{i}=\sum_{\alpha }Y_{i}^{\alpha }\;dX_{i}^{\alpha }.$$

Hence, each flux of extensity $J_{i}^{\alpha }$ carries an accompanying flux of energy given by

(3) $$I_{i}=Y_{i}^{\alpha }J_{i}^{\alpha }.$$

In the case of infinite reservoirs, their intensive quantities are used to describe their state which stay constant and do not change due to extensity or energy transfers to or from them.

Unlike reservoirs, the endoreversible subsystem type engine can store neither energy nor extensities. Hence, balance equations for extensities and energy have to be fulfilled at all time:

(4) $$0=\sum_{k}J_{i,k}^{\alpha }\;\mathrm{for}\;\mathrm{all}\;\alpha \;\mathrm{and}$$

(5) $$0=\sum_{k}I_{i,k},$$

where *k* consecutively numbers the contact points of subsystem *i* through which it is connected to other subsystems. The extensities of relevance in our case are entropy, volume and mole number.

These connections between the subsystems—called interactions—are defined either by equal intensities of the connected subsystems or by a specific transport law. In the first case, the occurring fluxes instantaneously equalize the intensities of the subsystems and may thus become infinitely large. However, when using transport laws, the phenomenological relationships typically ensure finite rates.

With the exception of entropy—since interactions can be irreversible and thus generate entropy—all extensities as well as energy have to be balanced over the interactions. Further, if it is not necessary to consider a certain specific type of extensity transferred via an interaction; a simple power flux can be used describing only the rate of energy or work transferred.

A more detailed description of the endoreversible formalism can be found, for example, in [20,43] and in [44], where the dissipative engine setup based on a more general interaction type is introduced in the latter. This dissipative engine setup is suitable to model engines for which efficiency or power output data are already given and may be arbitrary functions of any parameters. Here, this setup is considered as a black box model providing equations for loss fluxes and entropy production rates.

## 3. Model Description

**Figure 1:**
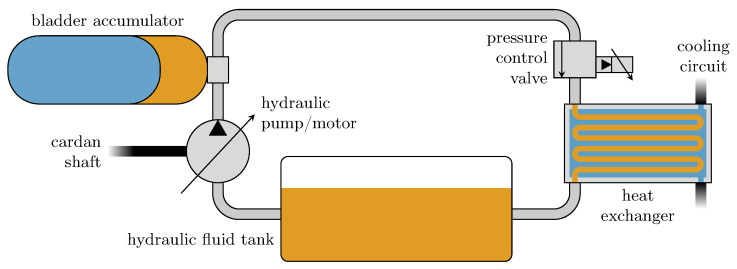
Scheme of the hydraulic recuperation system.

The hydraulic system consists of the following components: a hydraulic fluid tank, a hydraulic pump/motor which is mounted to and driven by the vehicle’s cardan shaft, a hydraulic bladder accumulator, a pressure control valve, a heat exchanger between the hydraulic fluid cycle and the vehicle’s cooling cycle and the pipes connecting named components. A scheme of the system is shown in Figure 1.

The hydraulic pump/motor, which we refer to as hydraulic unit, has the ability to act both as a pump and as a motor. This means that input power from the cardan shaft can be used to propel flow of hydraulic fluid, or a pressure driven fluid flow is converted to a power output put into the cardan shaft, respectively.

When energy is to be stored, the hydraulic unit works as a pump and hydraulic fluid is pumped from the low pressure hydraulic fluid tank towards the high-pressure bladder accumulator. The fluid compresses the gas inside the bladder of the accumulator leading to a pressure increase. The power needed to compress the gas is taken from the cardan shaft and hence the vehicle is decelerated. This supports the conventional disc brakes which therefore have to absorb less energy which results in reduced wear.

Once the gas is compressed and a certain amount of hydraulic fluid is in the bladder accumulator, the hydraulic unit can be operated as a motor. Now, a fluid flow in opposite direction generated by the gas pressure propels the hydraulic motor. Hence, the power carried by this fluid flow from a high to a low pressure is transferred to the cardan shaft accelerating the vehicle.

A pressure control valve is used to avoid that the pressure exceeds the maximum value suitable for the system. The application of this pressure control valve offers another interesting possibility: If the system has reached the maximum pressure, it can switch from the energy saving mode into a retarder mode. The pressure control valve then generates just enough fluid flow to maintain the pressure. Hence, the hydraulic unit can continue to operate and decelerate the cardan shaft. In this case, the pressure drop at the valve generates heat that warms the hydraulic fluid and the valve. The heat exchanger is used to cool the hydraulic fluid by transferring heat to the vehicle’s cooling circuit. This heat exchange might then be used to speed up the warming of the cooling circuit after a cold start so that optimal operation conditions are reached faster and a reduction of wear and fuel consumption can be achieved.

### 3.1. Hydraulic Fluid

To consistently incorporate pressure losses in the endoreversible description, a pressure-dependent equation of state able to model a fluid is needed. Here, the van der Waals equation is a suitable approach since it can map the pressure behavior of liquids quite well while being relatively easy to handle. More importantly, while being sufficiently accurate to examine general trends within the system, there is an explicit and analytically simple equation for the entropy of the van the Waals fluid, as shown below. The van der Waals equation can be written as

(6) $$\left(p+\frac{n^{2}a}{V^{2}}\right)\left(V-nb\right)=nRT,$$

with the van der Waals parameters *a* and *b*, and the universal gas constant *R*. Pressure, temperature and mole number of the fluid are denoted by *p*, *T* and *n*, respectively. The internal energy of the van der Waals fluid can be calculated as

(7) $$U={\hat{c}}_{V}nRT-\frac{an^{2}}{V},$$

with ${\hat{c}}_{V}$ being the dimensionless specific heat capacity at constant volume. Accordingly, the expression for the heat capacity results as

(8) $$C_{V}={\left(\frac{\partial U}{\partial T}\right)}_{V,n}={\hat{c}}_{V}nR.$$

To derive the internal energy of the van der Waals fluid as a function of its extensive quantities entropy, volume and mole number, which is the preferred description for endoreversible descriptions, we need the entropy of the fluid given by

(9) $$S\left(T,V,n\right)=nR\left({\hat{c}}_{V}\ln\frac{T}{T_{0}}+\ln\frac{V-nb}{V_{0}-n_{0}b}-\ln\frac{n}{n_{0}}\right)+n\frac{S_{0}}{n_{0}},$$

where $S_{0}\left(T_{0},V_{0},n_{0}\right)$ is a given reference state of the entropy at temperature $T_{0}$, volume $V_{0}$ and mole number $n_{0}$ [46]. Solving this equation for *T* results in

(10) $$T\left(S,V,n\right)={\left(\frac{\left(V_{0}-n_{0}b\right)T_{0}^{{\hat{c}}_{V}}}{n_{0}}\frac{n}{V-nb}\exp\left(\frac{S}{nR}-\frac{S_{0}}{n_{0}R}\right)\right)}^{\frac{1}{{\hat{c}}_{V}}}$$

which can be inserted into the equation for the internal energy, Equation (7). We then obtain the internal energy in dependence on the extensive quantities *S*, *V* and *n*

(11) $$U\left(S,V,n\right)={\hat{c}}_{V}nR{\left(\frac{\left(V_{0}-n_{0}b\right)T_{0}^{{\hat{c}}_{V}}}{n_{0}}\frac{n}{V-nb}\exp\left(\frac{S}{nR}-\frac{S_{0}}{n_{0}R}\right)\right)}^{\frac{1}{{\hat{c}}_{V}}}-\frac{an^{2}}{V}.$$

This equation has been called the principle equation of state by Essex and Andresen due to the fact that it captures all physical content in the relationship between the thermodynamic variables of the system [47]. Further, all other equations—which are typically referred to as equations of state—can be deduced from this expression. From the derivations of the principle equation of state with respect to the extensities *S*, *V* and *n*, we obtain the intensities

(12) $$T(S,V,n)={\left(\frac{\partial U}{\partial S}\right)}_{V,n}={\left(\frac{\left(V_{0}-n_{0}b\right)T_{0}^{{\hat{c}}_{V}}}{n_{0}}\frac{n}{V-nb}\exp\left(\frac{S}{nR}-\frac{S_{0}}{n_{0}R}\right)\right)}^{\frac{1}{{\hat{c}}_{V}}},$$

(13) $$p(S,V,n)=-{\left(\frac{\partial U}{\partial V}\right)}_{S,n}=\frac{nR}{V-nb}\;T\left(S,V,n\right)-\frac{an^{2}}{V^{2}},$$

(14) $$\mu (S,V,n)={\left(\frac{\partial U}{\partial n}\right)}_{S,V}=\left({\hat{c}}_{V}R+\frac{RV}{V-nb}-\frac{S}{n}\right)T\left(S,V,n\right)-\frac{2an}{V},$$

respectively, which are also functions of *S*, *V* and *n*.

Since there are no van der Waals parameters for the hydraulic fluid used in our application available—as well as parameters for more complex fluid equations such as Redlich–Kwong or Peng–Robinson—we have to determine these parameters using the known values of the coefficient of volumetric thermal expansion and the compressibility of the hydraulic fluid.

The coefficient of volumetric thermal expansion and the compressibility of a fluid are defined as

(15) $$\alpha_{V}=\frac{1}{V}{\left(\frac{\partial V}{\partial T}\right)}_{p},$$

(16) $$\beta =-\frac{1}{V}{\left(\frac{\partial V}{\partial p}\right)}_{T},$$

respectively. Solving the van der Waals equation for *p* and calculating its total differential, we obtain

(17) $$dp=\frac{nR}{V-nb}\;dT-\left(\frac{nRT}{{\left(V-nb\right)}^{2}}-\frac{2n^{2}a}{V^{3}}\right)dV.$$

By setting dp=0 we can derive the expression of the volumetric thermal expansion $\alpha_{V}$ of the van der Waals fluid as well as its compressibility β by setting dT=0 and we obtain

(18) $$\alpha_{V}=\frac{V-nb}{VT-\frac{2an}{R}{\left(\frac{V-nb}{V}\right)}^{2}},$$

(19) $$\beta =\frac{{\left(V-nb\right)}^{2}}{nRVT-2an^{2}{\left(\frac{V-nb}{V}\right)}^{2}}.$$

By inserting the values of the volumetric thermal expansion, the compressibility and the corresponding reference pressure and temperature into the above equations, we are now able to determine the parameters of the van der Waals equation, *a* and *b*.

What is missing for a complete description is the heat capacity of the fluid. Using Equation (8), the dimensionless specific heat capacity at constant volume of the van der Waals fluid can be expressed as

(20) $${\hat{c}}_{V}=\frac{C_{V}}{nR}.$$

Hence, for a given specific heat capacity at constant pressure $c_{V}=C_{V}/m$ and $m=nM=n\rho V_{m}$, where *M* is the molar mass and $V_{m}$ is the molar volume, the above equation can be written as

(21) $${\hat{c}}_{V}=c_{V}\frac{\rho V_{m}}{R},$$

where ρ is the density of the fluid. Since not the specific heat capacity at constant volume but constant pressure $c_{p}$ is usually given for liquids, we further need the relation between these two quantities given by

(22) $$c_{V}=c_{p}-\frac{\alpha_{V}^{2}T}{\rho \beta }.$$

Using the derived parameters we are able to model the physical properties of the hydraulic fluid as a van der Waals fluid.

### 3.2. Pipes and Hydraulic Fluid Tank

**Figure 2:**
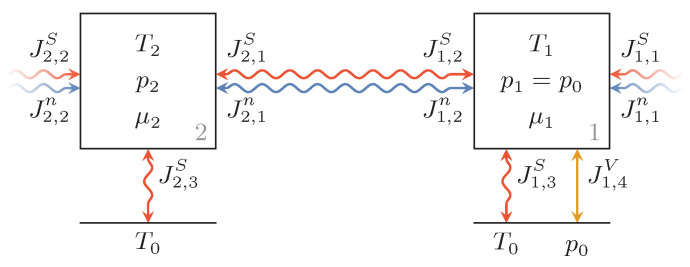
Endoreversible model of pipe segment (Reservoir 2) and hydraulic fluid tank (Reservoir 1) with combined particle and entropy fluxes between them and towards further pipe segments. Additionally, both reservoirs have irreversible heat transfers to the environment (line below), and the fluid tank is connected to the environment via a reversible volume flux.

The pipes of the recuperation system and the hydraulic fluid within the tank are modeled as reservoirs with constant volume and constant pressure, respectively. Figure 2 shows a pipe segment, Reservoir 2, and the hydraulic fluid tank, Reservoir 1. They are connected by a combined particle and entropy flux, and are coupled with other pipe segments as well, and have entropy fluxes to the environment (drawn as a line). Note that by "particle flow" the molar flow of the hydraulic fluid is meant. Since the volume of the fluid inside that tank can vary but its pressure is constant at ambient pressure, Reservoir 2 has an additional reversible connection transferring volume from or to the infinite environment reservoir.

We assume that the pipe segments do not run empty, but are always completely filled with hydraulic fluid and therefore the corresponding equations of state for the van der Waals fluid representing the hydraulic fluid apply with constant volume. To model pressure losses within the pipes, the volumetric flow rate *Q* of hydraulic fluid between the reservoirs is a function of the pressure difference Δp so that

(23) $$Q=Q\left(\Delta p\right)=V_{m}J^{n}\left(\Delta p\right)$$

with the molar volume $V_{m}$ and the particle flux $J^{n}$ holds.

To determine a suitable relation for that, we consider the Darcy–Weisbach equation expressing the relationship between pressure loss and mean flow velocity *u* as

(24) $$\Delta p=f_{D}\frac{\rho u^{2}}{2}\frac{l}{d_{i}},$$

where $f_{D}$, ρ, *l* and $d_{i}$ are the Darcy friction factor, the density of the fluid, and the length and the inner diameter of the pipe, respectively. The Darcy friction factor is given by

(25) fD,lam=64Re

for laminar flow and can be approximated using the Blasius correlation by

(26) fD,tur=0.316Re4

for a turbulent flow within the pipes, where Re is the Reynolds number.

For fluid flow in circular pipes, which is assumed here, the Reynolds number can be calculated as

(27) Re=udiν

and the mean flow velocity is given by

(28) $$u=\frac{Q}{A},$$

where ν and *A* are the kinematic viscosity of the fluid and the circular section of the pipe, respectively.

Since both laminar and turbulent flow are taken into account and numeric stability is ensured, the Reynolds number where the two Darcy friction factors given in Equations (25) and (26) are equal is used to define the transition between laminar and turbulent flow. This intersection lies at Reint=1189.39 and we can thus write

(29) $$\Delta p=\left\{\begin{array}{cc} \frac{32\;\rho \nu l}{d_{i}^{2}}u & \mathrm{if}\;u<u_{\mathrm{int}}\\ 0.158\;\rho \nu l\sqrt[{4}]{\frac{\nu u^{7}}{d_{i}^{5}}} & \mathrm{otherwise}, \end{array}\right.$$

where uint=Reintν/di is the corresponding mean flow velocity. From this, we can derive an expression for the mean flow velocity in dependence on pressure loss as

(30) u=di232ρνl(ΔpifΔp<ΔpintΔp0.158ρl4di5ν7otherwise,

with the pressure loss at the intersection point given by

(31) Δpint=32Reintρν2ldi3.

Using Equations (23) and (28), we can now write the resultant particle flux $J^{n}$ between two reservoirs as

(32) Jn=Adi232Vmρνl(Δpif|Δp|<Δpintsgn(Δp)AVmΔp0.158ρl4di5ν7otherwise,

where the molar volume $V_{m}$ depends on the temperature and pressure of the fluid. Thus, the particle flux between Pipe Segment 2 and Hydraulic Fluid Tank 1 can be written as

(33) $$J_{2,1}^{n}=J^{n}=-J_{1,2}^{n}$$

with $J^{n}$ from Equation (32) and $\Delta p=p_{1}-p_{2}$. As length *l*, we use the distance $l_{i,j}$ between two hydraulic components *i* and *j* which are connected by the pipe segment. The entropy flux that is coupled to the particle flux is simply defined by $J_{1,2}^{S}=S_{m,1}J_{1,2}^{n}$ for $p_{1}>p_{2}$ or $J_{2,1}^{S}=S_{m,2}J_{2,1}^{n}$ otherwise, where $S_{m,i}$ is the molar entropy of the *i*th subsystem.

Furthermore, we incorporate heat dissipation to the environment for which we assume a Newtonian heat transfer given by

(34) $$I_{2,3}=hl_{2}\left(T_{0}-T_{2}\right),$$

where *h* is the specific heat transfer coefficient per unit length and $l_{2}$ is the length of that pipe segment. To take the heat conduction through the pipe wall into account, we set

(35) $$h=\frac{\pi }{\frac{1}{k_{i}d_{i}}+\frac{1}{2\lambda }\ln\left(\frac{d_{o}}{d_{i}}\right)+\frac{1}{k_{o}d_{o}}}.$$

Here, λ, $k_{i}$, $k_{o}$, $d_{i}$ and $d_{o}$ are the thermal conductivity of the pipe wall, the inner and outer heat transfer coefficients, and the inner and outer diameter of the pipe, respectively.

The heat transfer from the hydraulic fluid within the tank to the environment is also assumed to be Newtonian and can be written as

(36) $$I_{1,3}=K_{1}\left(T_{0}-T_{1}\right),$$

where $K_{1}$ is an estimated overall heat transfer coefficient.

### 3.3. Hydraulic Unit

The hydraulic unit used in the recuperation system has the ability to act both as a hydraulic pump and as a hydraulic motor. The model for that hydraulic unit is a radial piston pump which has a movable floating eccentric ring around the pump shaft that determines the stroke of the pump’s pistons and thus the displacement of the hydraulic unit.

In this work, for simplicity, a displacement factor γ defining the ratio of current displacement to maximum displacement, so that −1≤γ≤1 holds, is used to describe the operation mode of the hydraulic unit. A negative displacement factor means that hydraulic fluid is pumped from the ambient pressure fluid tank to the high pressure bladder accumulator. A positive displacement factor means that a fluid flow from the high pressure bladder accumulator to the ambient pressure fluid tank generates a power output.

The hydraulic unit is mounted directly on the cardan shaft, which means that the cardan shaft actually is the shaft of the hydraulic unit. Since the unit does not move any pistons when the eccentric ring is in its neutral position and no clutch is needed, an additional moment of inertia is negligible, unlike in other hybrid systems such as those described in [8,9].

**Figure 3:**
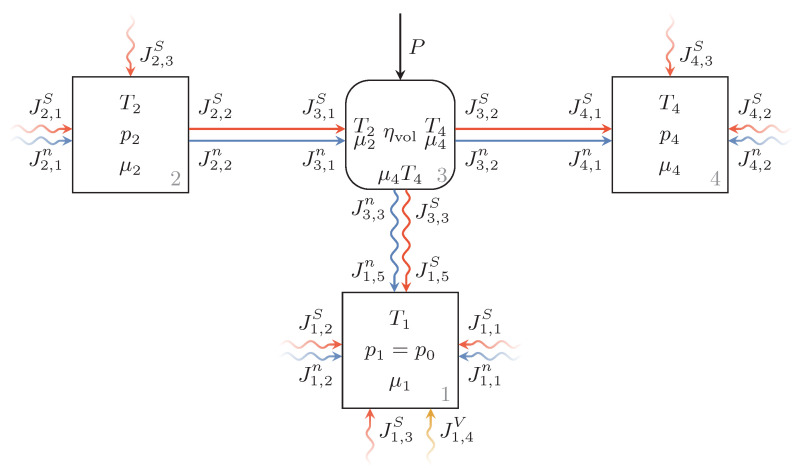
Endoreversible model of the hydraulic unit as a dissipative engine with given efficiency (Engine 3) and unspecified power input as well as hydraulic fluid tank and adjacent pipe segments (Reservoirs 1, 2 and 4, respectively). The flow directions are drawn according to the flow directions of the hydraulic unit operating in pump mode.

Figure 3 shows the endoreversible model of the hydraulic unit with adjacent pipe segments. Here, the flow directions are drawn according to the hydraulic unit acting as a pump, hence from Reservoir 2 to Reservoir 4. The hydraulic unit itself, Engine 3, is modeled as a dissipative engine (see [44]) with given volumetric efficiency $\eta_{\mathrm{vol}}$.

As mentioned above, the hydraulic unit can generate a variable volumetric flow rate *Q*. Depending on the displacement factor γ and the rotational speed of the shaft $n_{\mathrm{cyc}}$, this is given by

(37) $$Q=\gamma n_{\mathrm{cyc}}V_{d}\eta_{\mathrm{vol}}$$

where $V_{d}$ and $\eta_{\mathrm{vol}}$ are the maximum displacement and the volumetric efficiency of the unit. The input flow rate can thus be calculated using the above equation with $\eta_{\mathrm{vol}}=1$, while density differences between the low and high pressure side of the system can be neglected due to the low compressibility of the fluid. In the case of the pump mode, the incoming particle flux which is assumed to be reversible yields

(38) $$J_{2,2}^{n}=-J_{3,1}^{n}=\frac{\gamma n_{\mathrm{cyc}}V_{d}}{V_{m,2}},$$

where $V_{m,2}$ is the molar volume of Reservoir 2. The coupled entropy flux can be written as

(39) $$J_{2,2}^{S}=-J_{3,1}^{S}=S_{m,2}J_{2,2}^{n}$$

with $S_{m,2}$ being the molar entropy of Reservoir 2.

The chemical potentials and temperatures at the contact points towards these reservoirs are chosen to equal the intensive quantities of these reservoirs. As a consequence, Engine 3 transfers the combined particle and entropy flux from the state of the Reservoir 2 to the state of Reservoir 4. The necessary energy input for this conversion is given by the unspecified power transfer *P*.

The volumetric efficiency of the hydraulic unit defines the loss of particle and entropy transfer, or in other words the leakage of the operating hydraulic unit. This loss transfer in the case of the hydraulic unit in pump mode is given by

(40) $$J_{3,3}^{n}=\left(\eta_{\mathrm{vol}}-1\right)J_{3,1}^{n}\frac{V_{m,2}}{V_{m,4}},$$

(41) $$J_{3,3}^{S}=S_{m,2}J_{3,3}^{n},$$

where $V_{m,2}$ and $V_{m,4}$ are the molar volumes of Reservoirs 2 and 4, respectively. The entropy generated due to the loss flux results in a transfer of heated hydraulic fluid to Reservoir 1.

When the hydraulic unit is operating in the opposite direction, as a hydraulic motor, the above equations can be easily adapted. The interaction of Engine 3 and Reservoir 4 is then defining the influx of the hydraulic unit so that

(42) $$J_{4,1}^{n}=-J_{3,2}^{n}=-\frac{\gamma n_{\mathrm{cyc}}V_{d}}{V_{m,4}},$$

(43) $$J_{4,1}^{S}=-J_{3,2}^{S}=S_{m,4}J_{4,1}^{n},$$

holds, where $S_{m,4}$ is the molar entropy of Reservoir 4. Then, the loss transfer of the hydraulic unit in motor mode is defined as

(44) $$J_{3,3}^{n}=J_{3,2}^{n}\left(\eta_{\mathrm{vol}}-1\right),$$

(45) $$J_{3,3}^{S}=S_{m,4}J_{3,3}^{n}.$$

To incorporate the mechanical efficiency of the hydraulic unit $\eta_{\mathrm{mech}}$, in pump mode, we simply consider the input power transferred to the recuperation system $P_{\mathrm{rec}}$ from the cardan shaft to be given by

(46) $$P=P_{\mathrm{rec}}\eta_{\mathrm{mech}}.$$

In the case of the hydraulic unit acting as a motor, the power transferred to the cardan shaft is calculated as

(47) $$P_{\mathrm{rec}}=P\eta_{\mathrm{mech}}.$$

**Figure 4:**
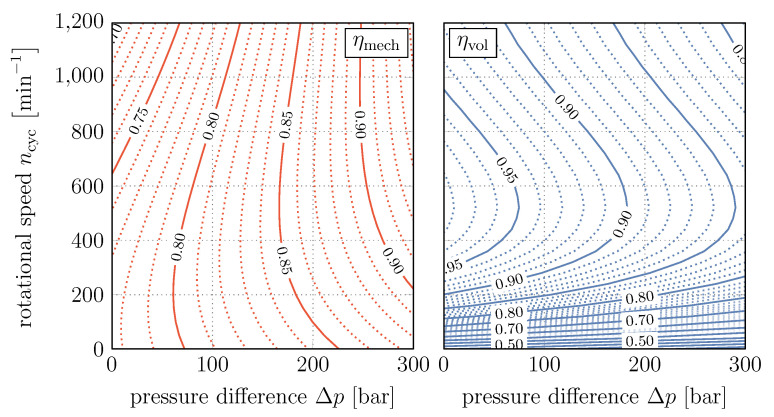
Mechanical and volumetric efficiency of the hydraulic unit over rotational speed and pressure difference for γ=0.5.

Furthermore, we use a realistic efficiency map which depends on the displacement factor γ, the rotational speed of the cardan shaft $n_{\mathrm{cyc}}$ and the difference of the applied pressures Δp. Hence, assuming the efficiencies to be identical for both directions of operation, for the mechanical and the volumetric efficiency applies

(48) $$\eta_{\mathrm{mech}}=\eta_{\mathrm{mech}}(|\gamma |,n_{\mathrm{cyc}},\Delta p),$$

(49) $$\eta_{\mathrm{vol}}=\eta_{\mathrm{vol}}(|\gamma |,n_{\mathrm{cyc}},\Delta p),$$

respectively. Figure 4 shows both efficiency maps over the relevant range of rotational speed and pressure difference for displacement factor γ=0.5.

The equations used for this are

(50) $$\eta_{\mathrm{mech}}=c_{1}n_{\mathrm{cyc}}+c_{2}\Delta p\left(c_{3}+c_{4}n_{\mathrm{cyc}}\right)+c_{5}{\left(n_{\mathrm{cyc}}+c_{6}\right)}^{3}+c_{7}\left|\gamma \right|n_{\mathrm{cyc}}+c_{8},$$

(51) $$\eta_{\mathrm{vol}}=c_{9}n_{\mathrm{cyc}}+c_{10}\ln\left(n_{\mathrm{cyc}}+1\right)+c_{11}(n_{\mathrm{cyc}}+c_{12}\left|\gamma \right|+c_{13}{)}^{3}+c_{14}\Delta p+c_{15}\left|\gamma \right|+c_{16},$$

with the parameters $c_{1}$ to $c_{16}$. These are not based on any hydraulic pump/motor model such as in [48], but are least squares fits on measured data of a real hydraulic unit with similar properties. Here, the use of the dissipative engine allows any conceivable efficiency characteristics without having to model the processes within the engine.

### 3.4. Bladder Accumulator

The bladder accumulator, which is used in the recuperation system to store brake energy, can be described as a cylinder with two chambers that are separated by a bladder totally enveloping one of the chambers. An inert gas within this bladder has a precharge pressure $p_{\mathrm{pre}}$, and the outside of the bladder is filled with hydraulic fluid and is connected to the hydraulic line. To prevent the bladder from being damaged when touching the outlet valve of the hydraulic fluid, a minimum volume of hydraulic fluid $V_{\min}$ has to remain within the accumulator. The bladder accumulator is typically characterized by its pressure range and its total volume $V_{\mathrm{acc}}$.

**Figure 5:**
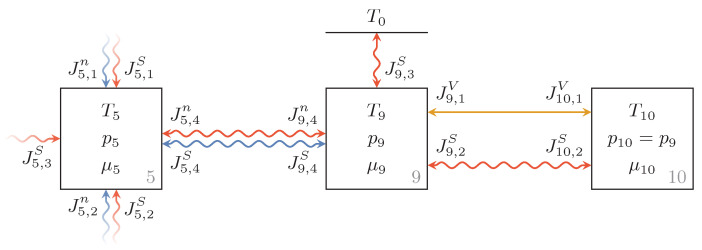
Endoreversible model of the bladder accumulator with adjacent pipe segment. Reservoirs 9 and 10 represent the hydraulic fluid and the gas within the bladder accumulator, respectively, which are connected via an irreversible heat transfer and a reversible volume flux. Further, there is an irreversible heat transfer from the hydraulic fluid to the environment.

The endoreversible model of this bladder accumulator and the adjacent pipe segment is shown in Figure 5. Reservoir 5 is the pipe segment that is connected to the accumulator and the Reservoirs 9 and 10 represent the hydraulic fluid, and the inert gas within the bladder, respectively. Since, within the accumulator, hydraulic fluid and inert gas have the same pressure $p_{9}=p_{10}$ and both share the total volume of the accumulator

(52) $$V_{\mathrm{acc}}=V_{9}+V_{10},$$

the two reservoirs representing them are reversibly connected by a volume transfer, where $J_{9,1}^{V}=-J_{10,1}^{V}$ holds. The inert gas itself which is assumed to be nitrogen is modeled as a van der Waals gas.

We assume that the bladder is always entirely surrounded by hydraulic fluid within the accumulator and can hence describe the thermal losses as heat transfers from the gas to the hydraulic fluid and from the hydraulic fluid to the environment. Modeled as Newtonian heat transfers these can be expressed as

(53) $$I_{9,2}=K_{\mathrm{acc}}\left(T_{10}-T_{9}\right)=-I_{10,2},$$

(54) $$I_{9,3}=K_{9}\left(T_{0}-T_{9}\right),$$

where $K_{\mathrm{acc}}$ and $K_{9}$ are the corresponding estimated heat transfer coefficients.

### 3.5. Pressure Control Valve

In this work, we restrict our investigations to the recuperation system’s function of drive support and model the pressure control valve as a simple pressure relief valve with a fixed control pressure and a high relief flux coefficient to avoid additional differential equations for valve control. In addition, for the sake of simplicity, we also refrain from modeling a heat exchanger and consider the valve housing as an additional reservoir that absorbs heat from the oil and releases it to the environment.

Since the pressure control valve simply transfers hydraulic fluid from a high pressure state to a low pressure state, and due to the fact that, unlike the hydraulic unit, there are no other energy conversions or transfers involved, an irreversible interaction is perfectly suitable to describe this process. The pressure relief through the valve is an isenthalpic process, which can be expressed by an energy conserving combined particle and entropy flux. The pressure drop that leads to an increase in internal energy during the isenthalpic process is expressed by the change in chemical potential and the generated entropy heating the fluid.

**Figure 6:**
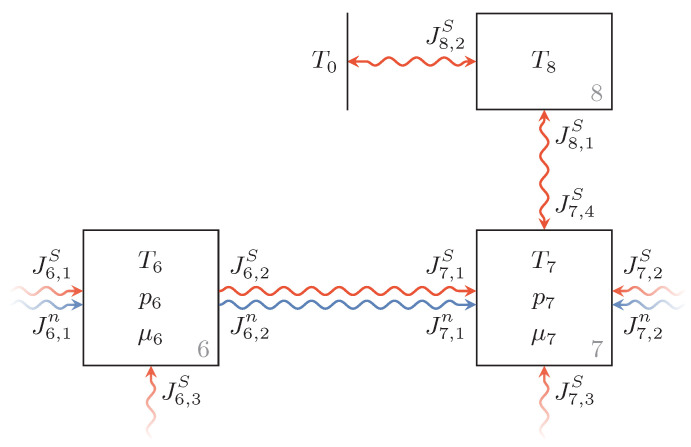
Endoreversible model of the pressure control valve represented by an irreversible combined particle and entropy flux between two pipe segments (Reservoirs 6 and 7). The valve housing (Reservoir 8) is connected to Reservoir 7 and the environment via irreversible heat transfers.

Figure 6 shows the endoreversible model consisting of the irreversible interaction which is between Reservoirs 6 and 7, as well as the valve housing represented by Reservoir 8, which is connected to Reservoir 7 via an irreversible heat transfer. The relief of hydraulic fluid through the valve is determined by

(55) $$J_{6,2}^{n}=\left\{\begin{array}{cc} \frac{\theta }{V_{m,6}}\left(\frac{p_{6}}{p_{\mathrm{valve}}}-1\right) & \mathrm{if}\;p_{6}>p_{\mathrm{valve}}\\ 0 & \mathrm{otherwise}, \end{array}\right.$$

where θ and $p_{\mathrm{valve}}$ are the relief flux coefficient and the relief pressure of the valve, respectively. The molar volume of the hydraulic fluid of Reservoir 6 is denoted by $V_{m,6}$ and its pressure by $p_{6}$.

The valve housing is modeled as a solid body whose entropy is given by

(56) $$S_{8}\left(T_{8}\right)=C_{p}\ln\frac{T_{8}}{T_{8,0}}+S_{8,0},$$

where $C_{p}$ is the heat capacity of the valve housing at constant pressure, and $T_{8,0}$ and $S_{8,0}$ are reference values for its temperature and corresponding entropy. Thus, the temperature of Reservoir 8 in dependence on the entropy can be expressed as

(57) $$T_{8}\left(S_{8}\right)=T_{8,0}\exp\left(\frac{S_{8}-S_{8,0}}{C_{p}}\right),$$

where we use $C_{p}=c_{p}m_{\mathrm{valve}}$ with the specific heat capacity of the valve housing material $c_{p}$ and its mass $m_{\mathrm{valve}}$. The heat transfer between the valve housing and the hydraulic fluid as well as that between the valve housing and the environment are assumed to obey Newton’s heat transfer law so that

(58) $$I_{8,1}=K_{\mathrm{valve}}\left(T_{7}-T_{8}\right)=-I_{7,4},$$

(59) $$I_{8,2}=K_{8}\left(T_{0}-T_{8}\right),$$

hold, with the estimated overall heat transfer coefficients $K_{\mathrm{valve}}$ and $K_{8}$, respectively.

### 3.6. Composite Model

**Figure 7:**
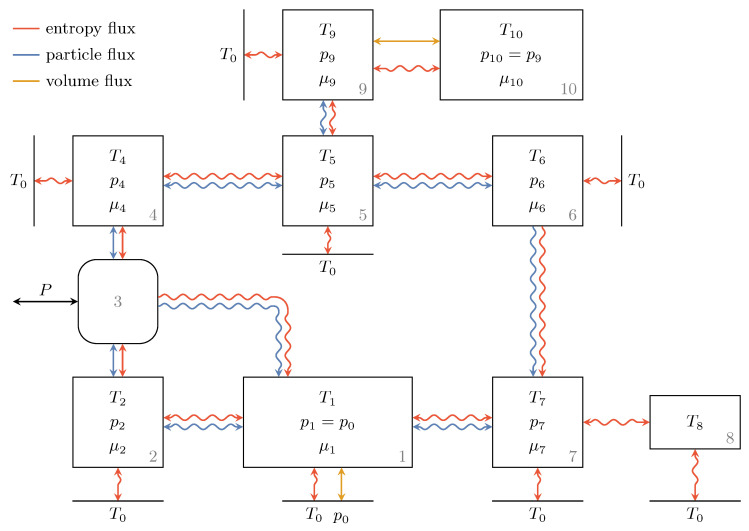
Composite endoreversible model of the recuperation system with the hydraulic components as described in the previous sections and the pipe segments represented by Reservoirs 2 and 4–7. Lines (instead of rectangles) represent the environment, and intensities as well as all incorporated fluxes are shown.

Having described the endoreversible models of all the individual components of the recuperation system, we can now take a look at the whole composite model shown in Figure 7. Five reservoirs, namely Subsystems 2, 4, 5, 6 and 7, are used to model the hydraulic pipes. The spatial resolution associated with that could be increased at will by increasing the number of reservoirs representing the pipes. The infinite environmental reservoir is again represented by lines, and the intensive quantities of all reservoirs are shown. Labeling the fluxes has been omitted for readability, but they are consistent with the descriptions of the previous sections. Here, the reservoirs above the hydraulic unit, Engine 3, represent the high pressure side and those below Engine 3 represent the low pressure side of the recuperation system. The dynamics are now described by the balance equations for the relevant extensities (entropy, volume and mole number) of all the respective subsystems. We exemplify this for Reservoir 8: the (only) relevant extensity is entropy and thus we have

(60) $$\begin{array}{cc} {\dot{S}}_{8} & =J_{8,1}^{S}+J_{8,2}^{S}=\frac{I_{8,1}+I_{8,2}}{T_{8}}\\ \; & =K_{\mathrm{valve}}\frac{T_{7}-T_{8}}{T_{8}}+K_{8}\frac{T_{0}-T_{8}}{T_{8}}, \end{array}$$

where $T_{8}$ has to be replaced by Equation (57) and so forth.

At this point, we would like to mention that all heat transfer parameters were estimated using the data and equations given in [49,50]. Other properties of the hydraulic components were estimated using product brochures [51,52,53].

### 3.7. Driving Dynamics of the Truck

If we are interested in the dynamical behavior of the system and possible energy savings, a given driving profile of a vehicle which can be equipped with the recuperation system provides a suitable basis for investigation. From such a driving profile we primarily need the velocity *v* of the vehicle and the height profile *h* or the angle of inclination α of the street as a function of time.

Using these variables, the power that is needed to accelerate or decelerate the truck can be expressed as

(61) $$P_{\mathrm{truck}}=f_{m}m\frac{dv}{dt}v+mgc_{\mathrm{rr}}\cos\left(\alpha \right)\left|v\right|+\frac{\rho_{\mathrm{air}}}{2}c_{d}A{\left|v\right|}^{3}+mg\sin\left(\alpha \right)v.$$

Here, the first term is the acceleration component, where $f_{m}$ and *m* are the mass factor and the mass of the vehicle, respectively. The second and third term represent the rolling resistance and aerodynamic drag of the vehicle, where *g* and $c_{\mathrm{rr}}$ are the gravitational acceleration and the coefficient of rolling resistance, respectively. The parameters of the third term $\rho_{\mathrm{air}}$, $c_{d}$ and *A* are the density of air, the drag coefficient and the cross sectional area of the truck. The last term of Equation (61) represents the change in potential energy at inclines.

If $P_{\mathrm{truck}}$ is positive, the truck has a power demand, which has to be provided by the recuperation system and the combustion engine

(62) $$P_{\mathrm{truck}}=P_{\mathrm{rec}}+P_{\mathrm{comb}}.$$

Accordingly, if $P_{\mathrm{truck}}$ is negative, power can be stored in the recuperation system or is transferred to the disc brakes where it is dissipated, and hence

(63) $$P_{\mathrm{truck}}=P_{\mathrm{rec}}+P_{\mathrm{brake}}.$$

For the comparison of energy consumption of the vehicle with and without the recuperation system, we consider the mass of the recuperation system $m_{\mathrm{rec}}$ as additional weight in Equation (61) with $m=m_{\mathrm{truck}}+m_{\mathrm{rec}}$ or $m=m_{\mathrm{truck}}$, respectively.

## 4. Energy Savings

We now have a look at the numerical results obtained with the composite endoreversible model of the hydraulic recuperation system. The dynamical behavior of the system and its individual components is discussed and a selection of parameters is varied to investigate their influence on the energy saving potential of the system.

For the initial state of the recuperation system, the temperatures are at ambient temperature and the bladder accumulator is at its precharge pressure with the minimum amount of hydraulic fluid $V_{\min}$ within.

We assume that the hydraulic system supports the conventional disc brakes while storing energy or being in retarder mode and acts as a drive support as soon as energy has been stored, and until $V_{\min}$ of the fluid is reached again. That means that for the given velocity and height profile we calculate the power demand or excess $P_{\mathrm{truck}}$ as given in Equation (61). For given $n_{\mathrm{cyc}}$, we then determine the displacement factor γ so that braking and acceleration are supported as much as possible.

**Figure 8:**
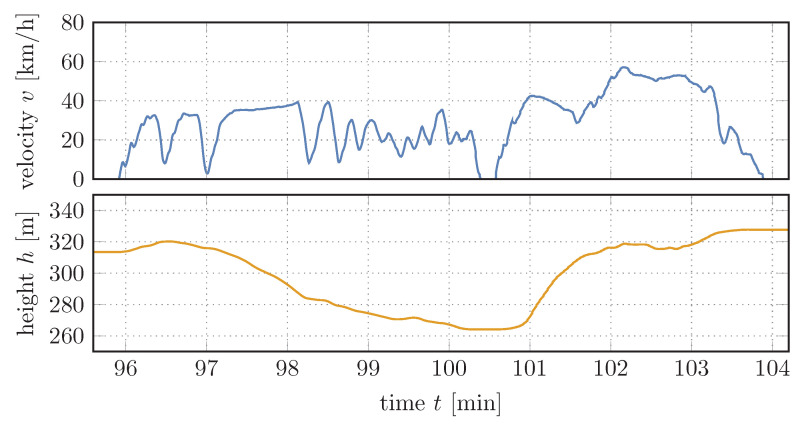
Section of investigated velocity and height profile over time.

**Figure 9:**
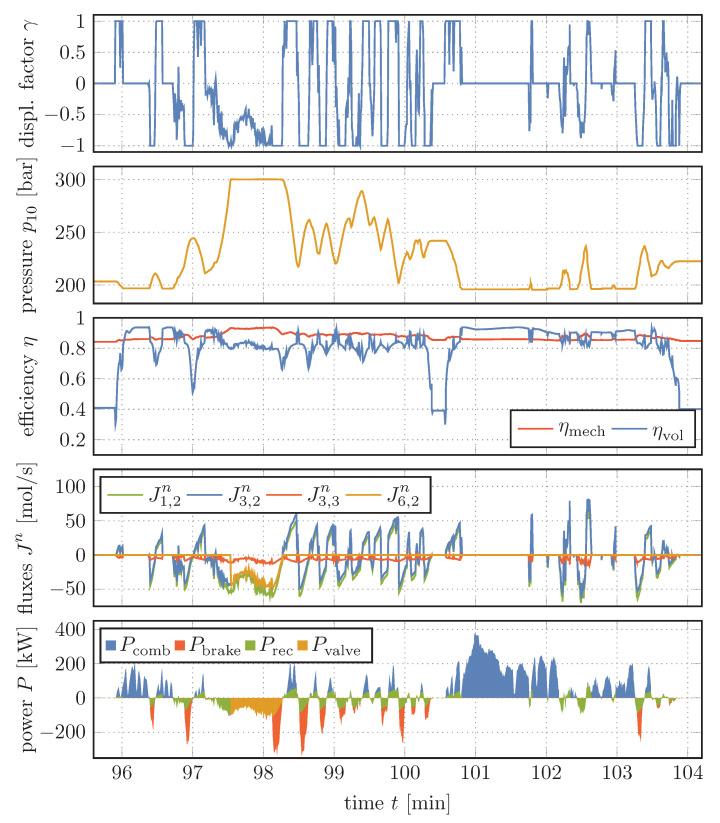
From top to bottom: Displacement factor, gas pressure, mechanical and volumetric efficiency of the hydraulic unit, selected particle fluxes and power shares over time (section).

**Figure 10:**
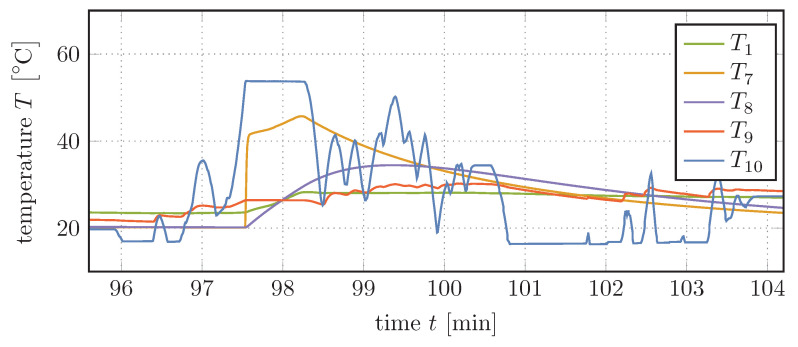
Temperatures of selected components over time (section).

A section of the velocity and height profile over time that will serve as basis for the calculations in this section is shown in Figure 8. The data used to create the 120-min profile were recorded on a truck collecting glass from bottle banks and therefore represents the typical daily operation and provides realistic results. Using this profile we can now simulate the dynamic behavior of the system, as shown in Figure 9 and Figure 10.

### 4.1. Dynamical Behavior of the System

Figure 9 shows the key variables describing the dynamic behavior of the system. At the top, we see the displacement factor which determines the generated hydraulic fluid flow which is pumped by or which propels the hydraulic unit at given pressure difference and rotational speed of the cardan shaft. A positive value stands for the fact that a hydraulic fluid flow from the bladder accumulator drives the hydraulic unit, which acts as a motor. This results in the falling pressures of the gas within the accumulator, which can be seen in the diagram below. In contrast, negative values indicate that the hydraulic unit is operating as a pump, transporting the hydraulic fluid from the tank to the high pressure side of the system. An increase of the pressure up to the relief value of the pressure control valve is the result.

In the middle of Figure 9, we can see the mechanical and volumetric efficiencies of the hydraulic unit, which depend on the displacement factor, rotational speed and applied pressure difference. While the mechanical efficiency remains relatively constant and appears to be largely determined by the pressure curve of the high pressure side of the system, the volumetric efficiency is subject to strong fluctuations, which are mainly related to the vehicle’s velocity and thus to the rotational speed of the cardan shaft. The latter determines the loss particle flux $J_{3,3}^{n}$ (see Equation (40)) to the hydraulic fluid tank, which is illustrated in red in the diagram below.

The fluxes $J_{1,2}^{n}$, $J_{3,2}^{n}$ and $J_{6,2}^{n}$ are the ones from the tank towards the hydraulic unit, from the hydraulic unit towards the bladder accumulator, and the particle flux through the pressure control valve, respectively. The signs of $J_{1,2}^{n}$ and $J_{3,2}^{n}$ thus correspond to the sign of the displacement factor and depend on the operation mode of the hydraulic unit. It can be seen that the amount of particle flux out of the engine (positive green, negative blue) is always decreased by the amount of the loss flux compared to the particle flux into the engine. Furthermore, around t=98min when the gas pressure has reached the relief pressure of the valve, the operation of the pressure control valve can be recognized. Here, $J_{3,2}^{n}$ and $J_{6,2}^{n}$ seem to be identical, which they are actually not due to a remaining minimal flow into the accumulator which we will come to later.

The bottom diagram shows the power shares which together give the total power $P_{\mathrm{truck}}$ needed to accelerate or decelerate the vehicle. In addition, the portion that could be achieved due to the use of the pressure control valve has been marked. Here, it can be seen that for 97min<t<100.5min large parts of the energy requirement of the truck can be covered by the recuperation system, whereas, e.g., from t=100.5min to around t=102min drive can hardly be supported. The reason for this difference is the velocity profile and, particularly in this case, the height profile. Until t=100.5min, the profile is slightly downhill, whereas it is steeply uphill after that. This results in the increased power demand of the truck that can only be covered by the combustion engine.

Figure 10 shows the temperatures $T_{1}$ and $T_{7}$–$T_{10}$, which belong to the hydraulic fluid tank, the pipe segment after the valve, the valve housing, and to the hydraulic fluid and gas within the bladder accumulator, respectively. It is particularly noticeable here how the temperature of the gas rises and falls with its pressure changes. At the same time, the temperatures of the hydraulic fluid within the bladder accumulator approaches the temperature of the gas. Particularly good to see is this exponential approach when—next to small interruptions—the amount of hydraulic fluid within the bladder accumulator remains constant after t=101min.

In contrast, the hydraulic fluid does not heat up but cools down around t=98min when the pressure control valve is active. This is due to the fact that the gas slowly gives off heat to the hydraulic fluid. The resulting decreasing volume of the gas provides space for more hydraulic fluid to enter the accumulator. This minimal remaining flux causes a slight cooling of the hydraulic fluid.

Meanwhile, the fluid flow through the pressure control valve heats up itself and thus also the valve housing, which slowly cools down to ambient temperature thereafter. Furthermore, the amount of heated hydraulic fluid that enters the tank slightly increases its temperature.

To obtain an estimate for the reduction in fuel consumption and brake wear, we look at the relative reduction in total energy that has to be provided by the combustion engine or absorbed by the disc brakes, respectively, during the whole driving cycle. In this example, savings of energy to accelerate the vehicle of $s_{\mathrm{ac}}=10.16\%$ could be achieved due to the use of the recuperation system. The braking energy, which has to be absorbed by the disc brakes is reduced by $s_{\mathrm{br}}=58.12\%$. Just as these values depend on the considered driving profile, they also depend on the chosen parameter values of the hydraulic recuperation system. In the next section, we deal with the variation of certain parameters and the consequences.

### 4.2. Variation of Selected Parameters

#### 4.2.1. Bladder Accumulator Volume and Displacement of Hydraulic Unit

**Figure 11:**
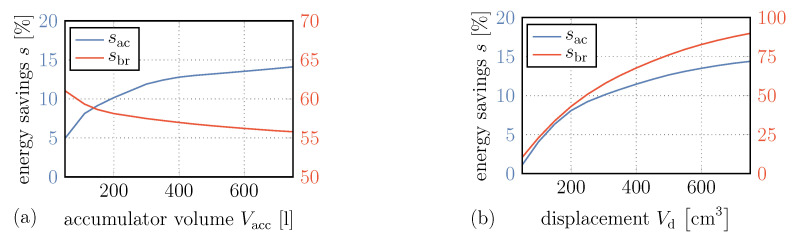
Energy savings both accelerating and decelerating the vehicle over bladder accumulator volume (**a**) and hydraulic unit displacement (**b**).

Energy savings accelerating and decelerating the truck, $s_{\mathrm{ac}}$ and $s_{\mathrm{br}}$, respectively, are shown over the bladder accumulator volume $V_{\mathrm{acc}}$ in Figure 11a. Here, we can see an increase in energy savings for acceleration with increasing accumulator volume. However, this increase is slightly reduced after $V_{\mathrm{acc}}=300\;l$. The reason for this is that with this volume already a large part of the braking processes are covered in the best possible way and the use of the pressure valve is reduced. A further increase in accumulator volume only helps in a few cases of long braking processes, such as the one at t=98min discussed above. The reusable amount of energy, however, does not increase for shorter braking processes.

If we look at the reduction in braking energy that has to be absorbed by the disc brakes, we even see a negative effect. Here, as the storage volume increases, the energy savings decrease. This is mainly due to the use of the pressure control valve, which allows continuing to support the braking process at relief pressure in retarder mode. Further, since a larger volume also causes a slower increase in pressure, the braking power, which can be approximated by

(64) P=QΔp

with the volumetric flow rate *Q* and the pressure difference Δp, is reduced.

Figure 11b shows the energy savings over $V_{d}$. In this case, for both acceleration and deceleration of the vehicle, an increase in the hydraulic unit’s displacement tremendously increases the energy savings. This is due to the fact that higher displacements generate higher volume flow rates at given rotational speeds and thus higher powers absorbed or provided by the recuperation system.

#### 4.2.2. Heat Transfer within the Bladder Accumulator

**Figure 12:**
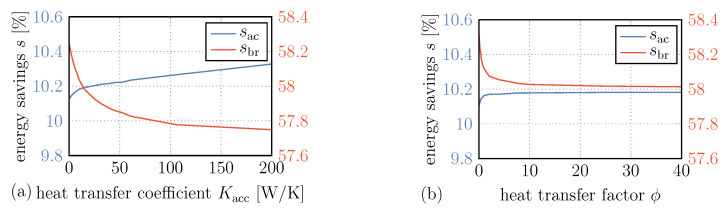
Energy savings both accelerating and decelerating the vehicle over heat transfer coefficient within the accumulator (**a**) and heat transfer factor (**b**). The latter scales the heat transfers of the pipes segments, the hydraulic fluid within the bladder accumulator and the hydraulic fluid tank to the environment.

The improvement of energy savings aside, the variation of parameters can also indicate the effects of loss factors such as heat and pressure losses. In Figure 12a, the energy savings over heat transfer coefficient $K_{\mathrm{acc}}$ is shown. This coefficient determines the heat transfer between the hydraulic fluid and the gas within the bladder accumulator.

One might assume that energy savings accelerating the vehicle decrease due to the decrease in gas pressure that results from the heat exchange with hydraulic fluid within the accumulator, and that this effect is enhanced at higher heat transfer coefficients. However, a contrary behavior can be observed and the lowest energy savings are obtained with zero heat transfer.

One reason for this is that, with decreasing pressure and hence decreasing volume of the gas within the bladder accumulator, there is—albeit very little—more space for hydraulic fluid to be pumped to the high pressure side of the recuperation system. In particular when the pressure control valve is active, as in the case discussed earlier, the amount of hydraulic fluid that can drive the hydraulic unit thereafter is increased while the high pressure remains.

Another reason for this effect is the heat transfer in the opposite direction, from the hydraulic fluid at ambient or even higher temperatures to the cold gas after expansion. This increases the gas pressure and thus the pressure on the high pressure side of the system, adding a small amount to the usable energy stored within the accumulator.

Note that this does not contradict the observations and explanations in [42], since the driving profile investigated here leads to substantially different effects than the driving profile with long waiting times investigated by Schwalbe et al.

Although the effects just mentioned seem to positively influence the amount of reusable energy, the effect of pressure loss outweighs them if we consider pure braking performance. Here, we can observe a decrease in energy savings $s_{\mathrm{br}}$ with increasing heat transfer coefficient, which is due to the lower power inputs with decreasing pressures.

#### 4.2.3. Heat Transfer to the Environment

The heat transfer of the hydraulic fluid in pipes, tank and bladder accumulator towards the environment should have an influence on the effect just observed. An increased heat transfer might result in lower temperatures of the hydraulic fluid entering the accumulator and enhance named effect. To investigate this, we varied the coefficients of the heat transfers from the pipe segments, the hydraulic fluid tank and the hydraulic fluid within the bladder accumulator towards the environment using a scaling factor ϕ. The resulting heat transfer coefficients are thus ϕ times the values used before.

The energy savings $s_{\mathrm{ac}}$ and $s_{\mathrm{br}}$ over this heat transfer factor are shown in Figure 12b. In fact, we can see that for increasing ϕ the energy savings during acceleration improve and the energy savings during deceleration decrease, and it can be assumed that the same reasoning applies here. However, in comparison to the variation of $K_{\mathrm{acc}}$, the magnitude of the effect is lowered and even approaches a limit here, and it can be assumed that this limit is determined by the heat transfer between the hydraulic fluid and gas within the accumulator.

#### 4.2.4. Pipe Diameter

**Figure 13:**
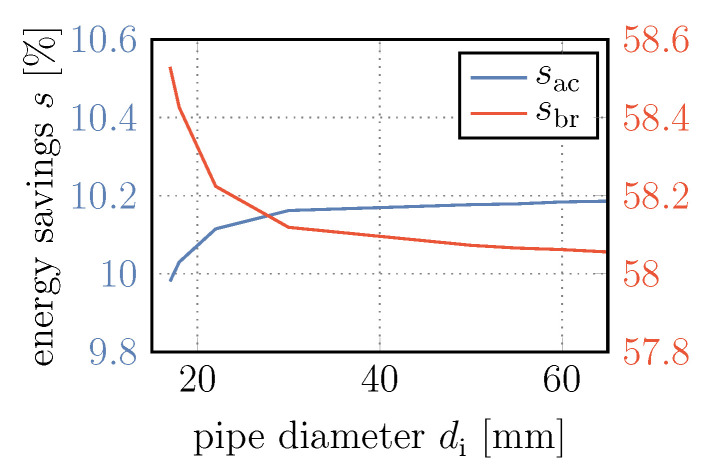
Energy savings both accelerating and decelerating the vehicle over inner pipe diameter.

Finally, we investigate the influence of the pipe diameter and thus the influence of the resulting pressure losses within the recuperation system. The energy savings over the inner diameter $d_{i}$ of the pipes can be seen in Figure 13. Without elaborating on Barlow’s formula, the outer pipe diameter is varied so that $d_{o}=1.1\;d_{i}$ holds. It should be noted that as the diameter of the pipe increases, so does the heat transfer of hydraulic fluid within the pipes to the environment, which might slightly enhance the effect of the hydraulic fluid cooling the gas.

Although the relative influence is much smaller here, the effect is similar to the that of the bladder accumulator volume. In the case of the hydraulic unit acting as a motor accelerating the vehicle, an increase in the inner pipe diameter leads to an increase in energy savings $s_{\mathrm{ac}}$, which asymptotically approach a maximum value. This maximum value corresponds to the pressure loss free ideal case. In contrast, in the case of the hydraulic unit in pump mode, the energy savings $s_{\mathrm{br}}$ decrease with increasing diameter. This is due to the fact that, as a result of the pressure losses, the pressure that the hydraulic unit has to overcome is higher than the pressure within the bladder accumulator or at the pressure control valve in retarder mode. According to Equation (64), this higher pressure generates higher power rates that can be received by the hydraulic recuperation system when braking and thus enhances the energy savings.

However, the pressure losses at the lower end of the plotted diameter range already cause negative pressures in the first pipe segment between the hydraulic fluid tank and the hydraulic unit when the latter is operating in pump mode. This is an indication that cavitation would occur at the hydraulic unit, permanently wearing out this component. Therefore, if smaller pipe diameters are used, a low pressure accumulator should be placed on the low pressure side of the hydraulic recuperation system instead of the hydraulic fluid tank, as can be seen in other publications [7,8].

## 5. Summary

In the present study, the endoreversible modeling of a hydraulic recuperation system was shown and explained. Both the working gas within the bladder accumulator and the hydraulic fluid are modeled as van der Waals fluids. In the latter case, the necessary van der Waals and caloric parameters were determined using the coefficient of volumetric thermal expansion, the compressibility and the specific heat capacity at constant pressure of the hydraulic fluid. Furthermore, pressure losses within the pipe segments were incorporated in the modeling for both laminar and turbulent flow regimes. Heat dissipation to the environment is taken into account for the hydraulic fluid tank, the pipes segments, the valve housing and the hydraulic fluid within the bladder accumulator using Newton’s heat law. Additionally, the heat transfer between the gas and hydraulic fluid within the bladder accumulator was incorporated. The modeling of the hydraulic unit was done using the dissipative engine with given efficiency introduced in [44]. The corresponding mechanical and volumetric efficiency maps were incorporated as nonlinear model fits on the basis of mapped data. After the endoreversible models of the individual components were introduced, the composite model of the recuperation system was shown and the power needed or provided by the truck when accelerating or decelerating, respectively, was calculated.

Using the velocity and height profile of recorded driving data from a vehicle suitable for the installation of the recuperation system, simulations of the dynamical behavior of the system were carried out to investigate the influence of certain parameters on the ability to save energy during acceleration and to reduce energy transferred to the disc brakes while braking. On a section of that driving profile, interesting effects in the temperature curves and fluid flows, especially when braking with active pressure control valve, were observed and explained. Overall energy savings during acceleration and deceleration around 10% and 58% can be achieved, respectively.

Subsequently, the parameters accumulator size and displacement of the hydraulic unit were varied to investigate and discuss their influence and potential for improving energy savings. Here, the energy savings accelerating the vehicle increased from less than 5% to almost 15% for either increasing accumulator volume or pump displacement. However, an increase in energy transferred to the conventional disc brakes up to 89.9% only occurred for higher pump displacement values. Using larger accumulator volumes decreased the effect from 61.1% to 55.8%. Finally, the coefficients of the heat transfer within the bladder accumulator and towards the environment, and the inner pipe diameter were varied to investigate the influence of their associated loss terms. In particular, the counterintuitive influence of the heat transfer within the bladder accumulator can be mentioned as an outstanding finding. Here, the lowest values were obtained with zero heat transfer and an increase by 0.5% was predicted with the highest tested heat transfer coefficient. The heat transfer to the environment accounts for changes of 0.1% and 0.4% at most regarding energy savings accelerating and decelerating the vehicle, respectively. A decrease of the pipe diameters led to a reduction in energy savings $s_{\mathrm{ac}}$ of up to 0.2% before negative pressures within the system indicate cavitation at pipe diameters below 18 mm.

## Nomenclature

α	inclination of the street	°
$\alpha_{V}$	coefficient of volumetric thermal expansion	k^−1^
β	compressibility	Pa^−1^
γ	displacement factor	-
θ	relief flux coefficient	m^3^ s^−1^
η	efficiency	-
λ	thermal conductivity	W K^−1^
μ	chemical potential	J mol^−1^
ν	kinematic viscosity	m^2^ s^−1^
ρ	density	kg m^−3^
*a*	cohesion pressure	Pa m^6^ mol^−2^
*A*	area	m^2^
*b*	co-volume	m^3^ mol^−1^
$c_{d}$	drag coefficient	-
$c_{p}$	specific heat capacity at constant pressure	J kg^−1^ K^−1^
$c_{\mathrm{rr}}$	coefficient of rolling resistance	-
$c_{V}$	specific heat capacity at constant volume	J kg^−1^ K^−1^
${\hat{c}}_{V}$	dimensionless heat capacity at constant volume	-
$C_{p}$	heat capacity at constant pressure	J K^−1^
$C_{V}$	heat capacity at constant volume	J K^−1^
$f_{D}$	Darcy friction factor	-
$f_{m}$	mass factor	-
*g*	gravitational acceleration	m s^−2^
*h*	specific heat transfer coefficient	W K^−1^ m^−1^
*I*	energy flux	W
*J*	extensity flux	*
*k*	heat transfer coefficient	W K^−1^ m^−2^
*K*	overall heat transfer coefficient	W K^−1^
*l*	length	m
*m*	mass	kg
*M*	molar mass	kg mol^−1^
*n*	mole number	mol
$n_{\mathrm{cyc}}$	rotational speed	s^−1^
*p*	pressure	Pa
*P*	power	W
*Q*	volumetric flow rate	m^3^ s^−1^
*R*	universal gas constant	J mol^−1^ K^−1^
Re	Reynolds number	-
*s*	energy savings	-
*t*	time	s
*T*	temperature	K
*u*	mean velocity of the fluid	m s^−1^
*U*	internal energy	J
*v*	velocity	m s^−1^
*V*	volume	m^3^
$V_{m}$	molar volume	m^3^ mol^−1^
$V_{d}$	displacement	m^3^
*X*	extensity	*
*Y*	intensity	*
* varying unit		
